# Surface proximity effect enables layer-by-layer growth of MoS_2_

**DOI:** 10.1093/nsr/nwac105

**Published:** 2022-06-06

**Authors:** Yang Chai

**Affiliations:** Department of Applied Physics, The Hong Kong Polytechnic University, China

Two-dimensional (2D) monolayer semiconductors have shown great potential for continuous downward scaling to a 2-nm technology node [1]. However, the monolayer characteristics make it quite challenging to completely manifest their intrinsic high performance. At the interface between semiconductors and dielectrics, the carriers are scattered by extrinsic impurities and remote optical phonons, which seriously degrade their carrier mobility; at the electrical contact, the relatively wide band gap of monolayer MoS_2_ hinders efficient carrier injection. Few-layer (bilayer or trilayer) MoS_2_ have been suggested with higher mobility and lower contact resistance, and can still retain excellent electrostatic control at sub-5-nm nodes [2]. However, precise control of growing wafer-scale bilayer or trilayer MoS_2_ remains a grand challenge from a thermodynamic perspective.

**Figure 1.**
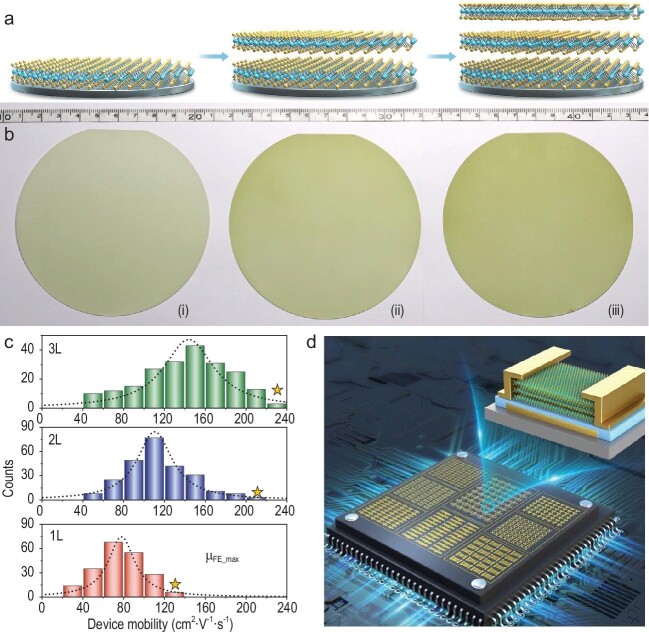
(a) Schematic illustration of the layer-by-layer growth process [5]. (b) Photographs of 4-inch (i) monolayer, (ii) bilayer and (iii) trilayer MoS_2_ wafers [5]. (c) Statistical distribution of the field-effect mobility of mono-, bi- and trilayer MoS_2_. The stars indicate the maximum values achieved in each type of device [5]. (d) Prospect of integrated logic circuits based on thicker-layer MoS_2_ wafers.

According to the criteria defined by E. Bauer and J.H. van der Merwe [3], the 2D growth mode requires that the substrate surface energy (*γ_s_*) is larger than the sum of freestanding MoS_2_ surface energy (*γ_o_*) and the MoS_2_/substrate interface energy (*γ_i_*), i.e. *γ_s_ > γ_o_ + γ_i_*. The surface energy of freestanding MoS_2_ increases with the number of layers, which results in the self-limiting characteristics of monolayer MoS_2_ growth. Different from the very recent edge-aligned bilayer strategy [4], Zhang and his colleagues analyze the surface proximity effect and successfully demonstrate controllable growth of wafer-scale bilayer and trilayer MoS_2_ in a layer-by-layer mode, by optimizing both thermodynamic and kinetic factors (Fig. 1a and b) [5].

Thermodynamically, they adopt sapphire (0001) with very high substrate surface energy (∼3.3 J/m^2^). By analyzing the energy relationship of MoS_2_/sapphire as a new substrate, they identify that it is still energetically feasible to grow additional MoS_2_ layers on monolayer and bilayer MoS_2_/sapphire substrate. This surface proximity effect enables the growth of bilayer and trilayer MoS_2_ in a layer-by-layer manner on MoS_2_/sapphire substrate with relatively high surface energy. With the increase of the layer number, the growth mode evolves from 2D to 3D because of the weakened proximity effect, which makes it thermodynamically unfavorable for growing thicker MoS_2_.

To achieve full coverage and a controlled layer number, researchers are also required to optimize kinetic growth factors. They increase Mo-source flux with high source temperature for high nucleation density on the substrate. By adopting oxygen-assisted chemical vapor deposition, they also achieve ultra-high edge growth rate (∼5–10 μm/min) compared to that without the oxygen assistance (<0.1 μm/min). With the optimization of the nucleation density and the edge growth rate, the diffusion mean free path is larger than the domain size, allowing uniform layer-by-layer growth at a wafer scale (Fig. 1b).

Furthermore, they fabricate long- and short-channel field-effect transistors using mono-, bi- and trilayer MoS_2_. Thick-layer field-effect transistors produce significant improvements in device performance. For long-channel devices (channel length of 5 to 50 μm), the average field-effect mobility is ∼80 cm^2^·V^−1^·s^−1^ for monolayers, to ∼110/145 cm^2^·V^−1^·s^−1^ for bilayer/trilayer devices (Fig. 1c). The high mobility of >100 cm^2^·V^−1^·s^−1^ uncovers the great potential of bilayer and trilayer MoS_2_ for high-performance transistors. For 100-nm short-channel devices, the current density increases with the layer number, i.e. 0.40 (monolayer), 0.64 (bilayer) and 0.81 (trilayer) mA/μm. Remarkably, the short-channel trilayer device with 40-nm channel length exhibits a record-high on-current density of 1.70 mA/μm at V_ds_ = 2 V and a high on/off ratio exceeding 10^7^. These device characteristics show high potential, with excellent electrostatic control and high drive current for end-of-roadmap transistors (Fig. 1d).

***Conflict of interest statement***. None declared.
